# An approach for medical event detection in Chinese clinical notes of electronic health records

**DOI:** 10.1186/s12911-019-0756-5

**Published:** 2019-04-09

**Authors:** Xuesi Zhou, Haoqi Xiong, Sihan Zeng, Xiangling Fu, Ji Wu

**Affiliations:** 1 Department of Electronic Engineering, Tsinghua University, Beijing, China; 2 Tsinghua-iFlytek Joint Laboratory, Beijing, China; 3 School of Software Engineering, Beijing University of Posts and Telecommunications, Beijing, China

**Keywords:** Medical event detection, Sequence labelling, Neural network models

## Abstract

**Background:**

Medical event detection in narrative clinical notes of electronic health records (EHRs) is a task designed for reading text and extracting information. Most of the previous work of medical event detection treats the task as extracting concepts at word granularity, which omits the overall structural information of the clinical notes. In this work, we treat each clinical note as a sequence of short sentences and propose an end-to-end deep neural network framework.

**Methods:**

We redefined the task as a sequence labelling task at short sentence granularity, and proposed a novel tag system correspondingly. The dataset were derived from a third-level grade-A hospital, consisting of 2000 annotated clinical notes according to our proposed tag system. The proposed end-to-end deep neural network framework consists of a feature extractor and a sequence labeller, and we explored different implementations respectively. We additionally proposed a smoothed Viterbi decoder as sequence labeller without additional parameter training, which can be a good alternative to conditional random field (CRF) when computing resources are limited.

**Results:**

Our sequence labelling models were compared to four baselines which treat the task as text classification of short sentences. Experimental results showed that our approach significantly outperforms the baselines. The best result was obtained by using the convolutional neural networks (CNNs) feature extractor and the sequential CRF sequence labeller, achieving an accuracy of 92.6%. Our proposed smoothed Viterbi decoder achieved a comparable accuracy of 90.07% with reduced training parameters, and brought more balanced performance across all categories, which means better generalization ability.

**Conclusions:**

Evaluated on our annotated dataset, the comparison results demonstrated the effectiveness of our approach for medical event detection in Chinese clinical notes of EHRs. The best feature extractor is the CNNs feature extractor, and the best sequence labeller is the sequential CRF decoder. And it was empirically verified that our proposed smoothed Viterbi decoder could bring better generalization ability while achieving comparable performance to the sequential CRF decoder.

## Background

EHRs cover a range of information associated with each patient encounter, including demographic information, medical history, immune status, laboratory findings, radiographic images etc. [1] In contrast to the structured portions of EHRs, clinical notes are more nuanced and are primarily used by healthcare providers for detailed documentation [2]. Narrative clinical notes of EHRs usually contain a wealth of information of medical events, including medication, indications, signs & symptoms, clinical examinations etc.

Medical event detection in narrative clinical notes of EHRs is a task designed for reading text and extracting information. A major challenge is the nature of unstructured and ’noisy’ text. We need to tackle the problems including incomplete sentences, irregular grammars, as well as abbreviations, medical terminology, and their synonyms.

Previous work usually treat the task as extracting concepts in text, which can be categorized as a Named Entity Recognition (NER) task, treating the text as a sequence of tokens [3–6]. However, these work mainly focus on the concepts at word granularity, such as drug names, adverse drug events (ADEs), indications, and attributes of these concepts. This manner omits the overall structural information of the medical record. In addition, due to the higher density of the Chinese language compared with English [7], the medical events in Chinese clinical notes usually covers several consecutive short sentences, leading to that medical event detection is more unsuitable at word granularity

To address these problems, we treat each clinical note as a sequence of short sentences, and correspondingly propose a novel tag system at short sentence granularity, paying attention to the overall information of clinical notes. Our tag system contains ten types of events, trying to model clinical notes from a perspective of higher semantic level while covering most of the medical-related information.

For the NER, which belongs to the sequence labelling tasks, the most widely used models mainly fall into two categories, statistics-based models and neural network-based models. Statistics-based models, such as Hidden Markov Models (HMM) and Conditional Random Fields (CRF), try to jointly infer the most likely label sequence for a given sentence. These models have been used with hand-crafted features and task-specific resources [8–10]. Beyond the above statistical models, the neural network-based models try to solve the problem without feature engineering. Collobert et al. [11] proposed an effective neural network model mainly using learned word embeddings trained on large quantities of unlabelled text as the input features, reducing the need for feature engineering. And with the development of deep learning, neural models have dominated many sequence labelling tasks, giving state-of-the-art results [12, 13]. In the clinical domain, neural network approaches, including CNNs and RNNs, have been applied to clinical event detection and concept extraction [4, 5, 14–16].

In this work, we treat our proposed medical event detection task as a sequence labelling task at short sentence granularity, and propose an end-to-end deep neural network framework requiring no task-specific resources and feature engineering. We handle the proposed task in a hierarchical way. We first use a feature extractor to get an embedding representation of each short sentence, taking the sequence at word granularity as input, and then obtain a sequence of embedding representations at short sentence granularity. Then we feed the short sentence level representations into a sequence labeller to jointly decode labels for the whole clinical note. We use convolutional neural networks (CNNs) [17] and bidirectional LSTM (bi-LSTM) as feature extractors, and use bi-LSTM and sequential CRF as sequence labellers. In addition, we propose a smoothed Viterbi decoder as sequence labeller, which can exploit the statistical information in the training corpus and decode label sequences without parameter training.

We also implement text classification models based on the CNNs feature extractor and bi-LSTM feature extractor respectively, which classify each short sentence independently, neglecting the correlation between short sentences. These models are used as baselines. We combine each feature extractor with each sequence labeller, and compare these models with the baselines.

We have collected and annotated 2000 clinical notes of EHRs from a third-level grade-A hospital according to our proposed tag system, and evaluate the models on this dataset. All our proposed models that combined feature extractor with sequence labeller outperform the baselines, verifying the effectiveness of the framework in capturing the overall information of clinical notes. The model consists of CNNs feature extractor and sequential CRF sequence labeller obtains the best performance, 92.6% accuracy for the sequence labelling of short sentences. The model using our smoothed Viterbi decoder obtains 90.7% accuracy, which is better than that of the bi-LSTM sequence labeller. Further, experiment results show that the smoothed Viterbi decoder brings better generalization ability while achieving good overall accuracy.

The contributions of this work are (i) redefining the task of medical event detection in Chinese clinical notes of EHRs and proposing a novel tag system of short sentence granularity. (ii) proposing a neural network framework for the redefined medical event detection task. (iii) giving empirical evaluations of this end-to-end framework on a dataset of 2000 annotated clinical notes. (iv) verifying the effectiveness of our proposed smoothed Viterbi decoder.

## Methods

### Dataset

Our dataset consists of 2000 Chinese clinical notes of EHRs, coming from a third-level grade-A hospital in China. We annotated the History of Present Illness Section of the clinical notes. By observing the data, we found that there are blocks consist of several consecutive short sentences as a semantic unit. For example, the four short sentences, ”, 39,, (there is fever on the next day, body temperature 39 degrees, no rash, no chills)”, integrally describe the symptoms of fever. If we only separately detect medical concepts such as “fever”, “body temperature”, “rash” and “chills”, it will destroy the semantic integrity and relevance of the short sentences. Therefore, we treat this text as a whole, a medical event called “Description of Symptoms”.

By referring to the writing specifications of clinical notes, we found that the writing of the history of present illness is basically in accordance with the order of symptom occurrence, symptom development, pre-hospital treatment, admission, general status. Therefore, we divided the medical events in clinical notes into 10 categories with higher levels of semantics compared to concepts such as drug names, including Description of Symptoms, History of Treatment, History of Diagnosis, Admission Status, General Status, Imaging Examination, Laboratory Examination, Electrocardiogram Examination, Endoscopy, and Pathological Examination. It basically covers all medical-related information in the History of Present Illness Section in clinical notes.

Each medical event consists of several consecutive short sentences with closely related semantics. The event categories and their examples are shown in Table 1.

**Table 1:** Medical event categories and examples

Category	Instance
Description of symptoms	There was no obvious cause of cough in the child for 6 days. The first cough was not severe, the next day there was fever, the body temperature was more than 39, no rash, no chills.
History of treatment	Taking retinoic acid induced differentiation therapy from 2014.4.30, 2014.5.3 using daunorubicin 40 mg×3 days chemotherapy
History of diagnosis	The patient found hbv infection 4 years ago
Admission status	Now I am seeking to further diagnose and treat the “coronary heart disease” to receive our hospital.
General status	During the course of the disease, the patient’s sleep diet may be normal, and the weight will not change significantly in the near future.
Imaging examination	Outpatient examination x-ray shows: right knee osteoarthritis
Laboratory examination	Outpatient OGTT examination showed fasting blood glucose 13.69 mmol/L, 2 hours blood glucose 23.77 mmol/L
Electrocardiogram examination	ECG prompts st-t segment depression, t-wave anomaly
Endoscopy	Electronic laryngoscopy: right vocal polyp
Pathological examination	Lively cut a piece to send pathology to show chronic inflammation of the rectal mucosa with polypoid hyperplasia

The 2000 clinical notes were annotated by medical students with more than 5 years of learning experience. Short sentences (with commas and periods as separators) were the smallest unit of annotation, and each short sentence belongs to at most one category. The labels consists of 10 medical events and 1 label “Others” representing non-medical events or other medical events, totalling 11 categories. We divided the 2000 annotated clinical notes into training, development, and test sets at a ratio of 7:1.5:1.5. The distribution of 11 categories in the dataset is shown in Table 2.

**Table 2:** The distribution of 11 labels in the dataset

	Train	Dev.	Test
Description of symptoms	13633	2921	2881
History of treatment	1845	341	371
History of diagnosis	485	59	105
Admission status	3071	672	653
General status	7100	1475	1446
Imaging examination	1468	389	238
Laboratory examination	1114	260	211
Electrocardiogram examination	41	21	16
Endoscopy	19	6	7
Pathological examination	128	53	25
Others	1927	384	424
Total	30831	6581	6377

### Baselines models

It is most intuitive to treat the problem as a text classification problem of short sentences. We implemented several baseline models treating the task as text classification of short sentences. According to [18], the text-CNN model has achieved excellent performance in sentence classification task. We implemented the text-CNN model using convolution kernels of different lengths to perform one-dimensional convolutions on the word embeddings of the input short sentences, obtaining the feature representations by max-pooling, and finally classifying the short sentences by a fully connected softmax layer.

In addition to the text-CNN model, we also implemented models based on bi-LSTM of different layers as our baselines.

### Sequence labelling models

However, as shown in Table 1, each event may consist of several consecutive short sentences, indicating the correlation that exists between the short sentences. The way treating the problem as a classification problem ignores this correlation and may cause the loss of contextual information, resulting in a decrease in accuracy of medical event detection.

In order to introduce correlation between short sentences into the model, we redefined the task as sequence labelling at short sentence granularity. And we proposed an end-to-end deep neural network consists of a feature extractor and a sequence labeller. We extract the semantic features of each short sentence, and then use these features as the input for the sequence labeller to obtain the optimal label sequence.

#### Feature extractor

Following the Baselines, we implemented feature extractors based on CNNs and bi-LSTM respectively.

#### Sequence labeller

We implemented sequence labellers based on sequential CRF and bi-LSTM respectively, which are the most commonly used decoders of the state-of-the-art sequence labelling models.

In addition, we proposed a smoothed Viterbi decoder, which combines the classification probability distributions of short sentences and the transition probability matrix of medical event categories to make predictions.

Specifically, we separately calculate the conditional probability distribution of the labels of the next short sentence according to the corpus, under the condition that the current short sentence belongs to each category. We take these distributions as the global transition probability matrix. And for each clinical note, treated as a sequence of short sentences, the feature representations of short sentences obtained by the feature extractor are passed to a fully connected softmax layer whose output are the context-independent classification probability distribution of the input short sentence. Finally, using the classification probability distributions and the transition probability matrix, it can be decoded by the Viterbi algorithm to obtain the optimal label sequence of the clinical note.

However, it should be noted that the above classification probability distributions and transition probability matrix come from different perspectives. The former is determined by the parameters of the neural network model, while the latter is determined by the annotation of the dataset, resulting in inconsistencies between them. We introduce an exponential smoothing factor for the transition probability to mitigate the impact of this problem.

Let *T*_*ij*_ be the transition probability from label *i* to label *j*, and the smoothed transition probability is

$$ \widehat{T}_{ij} = \frac{T^{c}_{ij}}{\sum_{k}T^{c}_{ik}} $$

Where *c* is the exponential smoothing factor, and we set it to 0.3 according the experimental results.

## Results

The experiments are divided into two parts. First, we evaluated the performance of the baselines, treating the task as a classification problem of short sentences. Then we evaluated the performance of our sequence labelling models, treating the task as a sequence labelling problem of short sentences.

### Experiment setting

#### Data

As described above, we divided the 2000 annotated clinical notes into training, development, and test sets at a ratio of 7:1.5:1.5. We segmented the sentences with our Chinese word segmentation tool, and used the word2vec tool to pre-train the word embeddings of 100 dimensions. The word embeddings were fine-tuned during training.

#### Model implementation and hyper-parameters

For the CNNs used in the text-CNN baseline and the feature extractor, we use ReLU as the non-linear unit and convolution kernels of 3, 4, 5 with 100 feature maps each. For the bi-LSTMs used in the text classification baseline, feature extractor and sequence labeller, we set the state size of bi-LSTM to 128. We used bi-LSTMs of 1 layer in the sequence labeller. And for the bi-LSTMs used in the text classification baseline and feature extractor, we experimented with 1, 2, and 3 layers, respectively. To mitigate the overfitting problem, we applied the dropout method [19] in the CNNs and bi-LSTMs to regularize our models, setting the dropout rate to 0.5. We trained our models with Adam optimizer [20], using the learning rate of 0.001.

#### Evaluation metrics

All results in the experiments were evaluated by accuracy.

### Results of text classification models

In this part, we take the label with the highest context-independent classification probability distribution as the prediction of each short sentence. The performance of this classification experiment is shown in Table 3. As expected, the text-CNN consistently outperforms the bi-LSTM based models, verifying the effectiveness of CNNs in feature extraction of sentences, especially short sentences.

**Table 3:** Medical event detection accuracy of our classification models on the test set

Model	Acc.
bi-LSTM(1 layer)	82.4%
bi-LSTM(2 layers)	84.2%
bi-LSTM(3 layers)	83.1%
**text-CNN**	**85.8%**

The bold font means the best result

Among the bi-LSTM based models, the 1 layer bi-LSTM model was the worst, and the 2 layer bi-LSTM model is the best among the bi-LSTM based models, but the accuracy is still 1.6% lower than that of the text-CNN model.

### Results of sequence labelling models

The performance of the sequence labelling models is shown in Table 4.

**Table 4:** Medical event detection accuracy of our sequence labelling models on the test set

	Model	Acc.
Text classification models	bi-LSTM(1 layer)	82.4%
	bi-LSTM(2 layers)	84.2%
	bi-LSTM(3 layers)	83.1%
	text-CNN	85.8%
Sequence labelling models	bi-LSTM(2 layers) feature extractor + bi-LSTM decoder	87.3%
	bi-LSTM(2 layers) feature extractor + smoothed Viterbi decoder	87.4%
	bi-LSTM(2 layers) feature extractor + CRF decoder	92.1%
	CNNs feature extractor + bi-LSTM decoder	89.9%
	CNNs feature extractor + smoothed Viterbi decoder	90.7%
	**CNNs feature extractor + CRF decoder**	**92.6%**

The bold font means the best result

The performance of the sequence labelling models is significantly higher than that of the text classification models, which indicates that the sequence labelling models can effectively exploit the context information to improve the accuracy, and verifies the correlation that exists between the short sentences.

Note that, consistent with results of text classification, the CNNs based feature extractor outperforms the bi-LSTM based ones, further verifying the effectiveness of CNNs in feature extraction of sentences.

## Discussion

### bi-LSTM vs. the smoothed Viterbi vs. sequential CRF

Because the transition probability matrix in the smoothed Viterbi decoder and the sequential CRF decoder introduces global information of the corpus, the performance of these two decoders is better than that of the bi-LSTM decoder which only incorporates the information of each sentence. Regardless of the feature extractor combined with, the sequential CRF decoder outperforms all the other decoders. Meanwhile, without additional parameter training for the decoder, the performance of the smoothed Viterbi decoder is between that of the sequential CRF decoder and the bi-LSTM decoder, indicating that the smoothed Viterbi decoder is a good alternative in the case of limited computing resources.

### Effect of the smoothing factor *c*

**Fig. 1:**
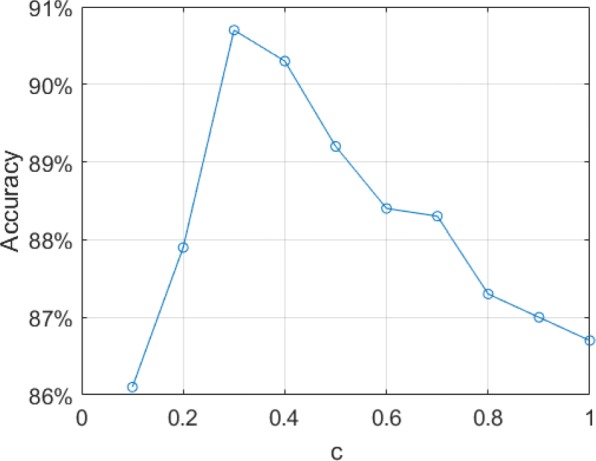
Effect of the smoothed factor *c*. The points denote the performance of the CNNs feature extractor+smoothed Viterbi decoder model on the test set. The optimal performance is obtained when *c*=0.3

As mentioned earlier, in order to solve the problem that there are inconsistencies between the classification probability distribution and the transition probability matrix, we introduce the exponential smoothing factor *c* for the transition probability matrix. The role of *c* is reflected in the weakening of the influence of the transition probability matrix on the predictions. When *c*=0, the transition probability distribution of all categories will become uniform distribution, the smoothed Viterbi decoder will lose the ability to sequence modelling, and the model will degenerate into the classification model, outputting the label with the highest classification probability directly. When c is close to 1, the transition probability matrix will dominate the sequence modelling, resulting in the negligence of the classification probability distribution, which also brings performance loss. Effect of *c* for the performance on the test set is shown in Fig. 1, and the optimal performance is obtained when *c*=0.3.

### Error analysis

**Fig. 2:**
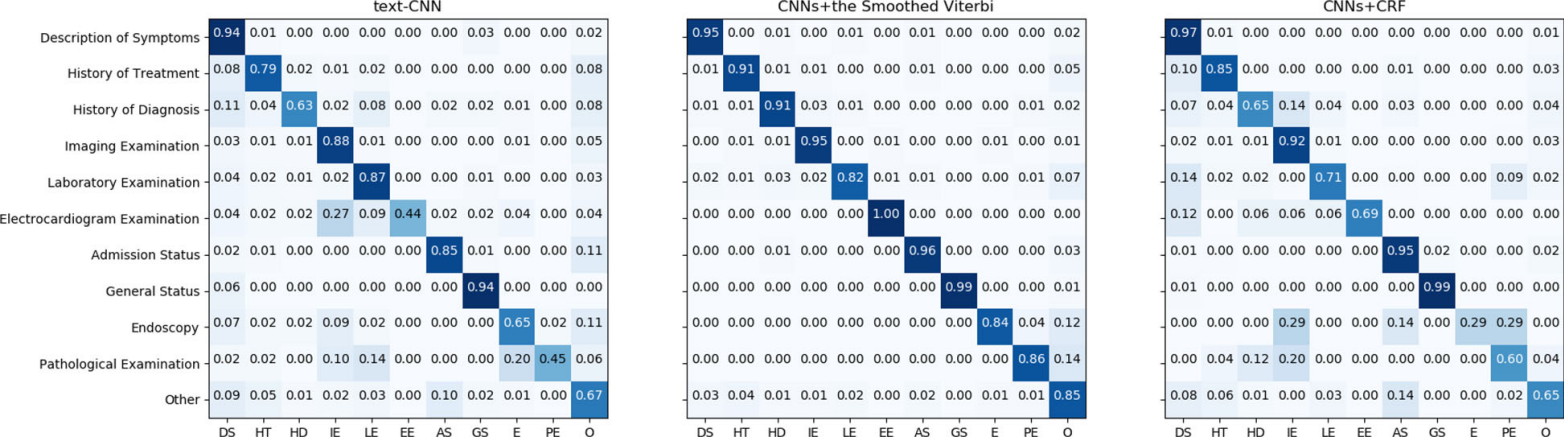
Heat-maps of confusion matrices. The three confusion matrices correspond to the text-CNN model, the CNNs feature extractor+the smoothed Viterbi decoder and the CNNs feature extractor+the sequential CRF decoder, respectively. Rows are reference and columns are predictions. The value in cell (*i*,*j*) denotes the percentage of short sentences in label *i* that were predicted as label *j*

We selected three models for error analysis, which are the text classification model of text-CNN, the sequence labelling model of CNNs feature extractor+the smoothed Viterbi decoder and the sequence labelling model of CNNs feature extractor+the sequential CRF decoder. The heat-maps of matrices of these models are shown in Fig. 2.

The first observation is that although the the performance of the CNNs+the smoothed Viterbi is lower than that of the CNNs+CRF in terms of overall accuracy, it is more balanced across all categories, compared to the other two models. The CNNs+CRF outperforms the CNNs+the smoothed Viterbi only in the category of “Description of Symptoms”, which has the largest number of samples. Due to the more trainable parameters of the sequential CRF decoder, the model is better fitted to the category of “Description of Symptoms”, but at the same time it makes the model more susceptible to the data bias of the dataset. This observation shows that our proposed smoothed Viterbi decoder brings better generalization ability while achieving good overall accuracy.

Another observation is that the accuracy of each medical event category is closely related to the distribution of labels on all the models. Although the the CNNs+the smoothed Viterbi performs fairly balanced in each category, the categories of the worst performance are the categories with the fewest samples, except for Laboratory Examination and Others, which are the categories with the most “noisy” text. For example, the accuracy of Endoscopy and Pathological Examination whose samples account for less than 1% is less than 87%.

## Conclusions

In this work, we have redefined the task of medical event detection in Chinese clinical notes of EHRs and proposed a novel tag system of short sentence granularity. Through the conversion from word level to short sentence level for the sequence labelling task, we could capture higher-level semantic events in clinical notes and obtain the global structural information. For the redefined task, we have proposed an end-to-end neural network framework consists of a feature extractor and a sequence labeller. In the sequence labelling part, in addition to implementing two common methods of CRF and bi-LSTM, we also have proposed the smoothed Viterbi decoder, a sequence labeller based on statistical information, that requires no additional parameter training.

We have annotated the History of Present Illness Section of 2000 clinical notes from a third-level grade-A hospital in China and conducted a series of experiments on this dataset.

Our results show that the sequence labelling models consistently outperform the classification models. And among the sequence labelling models, the best feature extractor is the CNNs feature extractor, and the best sequence labeller is the sequential CRF decoder. In addition, the results empirically verify that our proposed smoothed Viterbi decoder brings better generalization ability while achieving comparable performance to the sequential CRF decoder.

## References

[1] Birkhead GS, Klompas M, Shah NR (2015). Uses of electronic health records for public health surveillance to advance public health. Annu Rev Public Health.

[2] Shickel B, Tighe PJ, Bihorac A, Rashidi P, Deep EHR (2018). A survey of recent advances in deep learning techniques for electronic health record (EHR) analysis. IEEE J Biomed Health Inf.

[3] Jagannatha AN, Yu H (2016). Structured prediction models for RNN based sequence labeling in clinical text. Proceedings of the 2016 Conference on Empirical Methods in Natural Language Processing.

[4] Jagannatha AN, Yu H (2016). Bidirectional RNN for medical event detection in electronic health records.

[5] Wu Y, Jiang M, Lei J, Xu H (2015). Named entity recognition in Chinese clinical text using deep neural network. Stud Health Technol Inf.

[6] Xu D, Zhang M, Zhao T, Ge C, Gao W, Wei J (2015). Data-driven information extraction from Chinese electronic medical records. PLoS ONE.

[7] Hoosain R (2013). Psycholinguistic implications for linguistic relativity: A case study of Chinese.

[8] Luo G, Huang X, Lin CY, Nie Z (2015). Joint entity recognition and disambiguation. Proceedings of the 2015 Conference on Empirical Methods in Natural Language Processing.

[9] Passos A, Kumar V, McCallum A (2014). Lexicon infused phrase embeddings for named entity resolution. Proceedings of the Eighteenth Conference on Computational Natural Language Learning.

[10] Ratinov L, Roth D (2009). Design challenges and misconceptions in named entity recognition. Proceedings of the Thirteenth Conference on Computational Natural Language Learning.

[11] Collobert R, Weston J, Bottou L, Karlen M, Kavukcuoglu K, Kuksa P (2011). Natural language processing (almost) from scratch. J Mach Learn Res.

[12] Lample G, Ballesteros M, Subramanian S, Kawakami K, Dyer C (2016). Neural architectures for named entity recognition. Proceedings of the 2016 Conference of the North American Chapter of the Association for Computational Linguistics: Human Language Technologies.

[13] Ma X, Hovy E (2016). End-to-end sequence labeling via bi-directional LSTM-CNNs-CRF. Proceedings of the 54th Annual Meeting of the Association for Computational Linguistics (Volume 1: Long Papers).

[14] Li P, Huang H. Clinical information extraction via convolutional neural network. arXiv preprint arXiv:1603.09381.2016. https://arxiv.org/abs/1603.09381.

[15] Tutubalina E, Nikolenko S (2017). Combination of deep recurrent neural networks and conditional random fields for extracting adverse drug reactions from user reviews. J Healthc Eng.

[16] Viani N, Miller TA, Dligach D, Bethard S, Napolitano C, Priori SG, et al.Recurrent neural network architectures for event extraction from Italian medical reports. In: Artificial Intelligence in Medicine. AIME 2017. Cham: 2017. p. 198–202.

[17] LeCun Y, Boser B, Denker JS, Henderson D, Howard RE, Hubbard W (1989). Backpropagation applied to handwritten zip code recognition. Neural Comput.

[18] Kim Y. Convolutional neural networks for sentence classification. In: Proceedings of the 2014 Conference on Empirical Methods in Natural Language Processing. The Association for Computational Linguistics: 2014. p. 1746–51.

[19] Srivastava N, Hinton G, Krizhevsky A, Sutskever I, Salakhutdinov R (2014). Dropout: A simple way to prevent neural networks from overfitting. J Mach Learn Res.

[20] Kingma DP, Ba J. Adam: A method for stochastic optimization. arXiv preprint arXiv:14126980. 2014. https://arxiv.org/abs/1412.6980.

